# Primary laryngeal aspergillosis in the immunocompetent state: a clinical update^☆^

**DOI:** 10.1016/j.bjorl.2015.06.002

**Published:** 2015-10-17

**Authors:** Mainak Dutta, Arijit Jotdar, Sohag Kundu, Bhaskar Ghosh, Subrata Mukhopadhyay

**Affiliations:** Medical College and Hospital, Department of Otorhinolaryngology and Head-Neck Surgery, Kolkata, West Bengal, India

## Introduction

Laryngeal aspergillosis is known to occur in immunocompromised states, particularly in diabetes mellitus, tuberculosis, and human immuno-deficiency virus (HIV) infection, and is associated with use of inhalational steroids and cytotoxic drugs. Primary laryngeal aspergillosis is rare, especially in immunocompetent patients, with very few reported cases to date. It often mimics the pre-malignant and malignant conditions of larynx, and responds well to antifungals. This report presents a case of primary laryngeal aspergillosis in an immunocompetent middle-aged woman, and explores the current pool of evidence regarding its pathogenesis and clinical aspects. To date, this represents the only comprehensive review on the topic.

## Case report

A 45-year-old woman presented with progressive hoarseness for two months. It was preceded by an episode of sore throat which subsided with medication. There was no history of difficulty in deglutition, respiratory distress, and voice abuse. There was no recent-onset loss of weight and appetite, with no cough or evening rise in temperature. Neither she nor any of her kin had history of pulmonary tuberculosis. She was not addicted to tobacco or alcohol, and was otherwise healthy.

**Figure 1:**
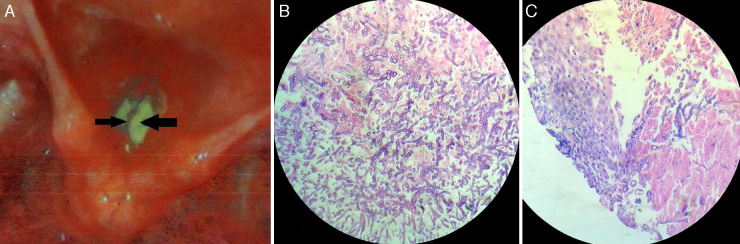
(A) Fiber-optic laryngoscopy revealed inflamed vocal cords covered with dirty white necrotic debris (arrows) that resembled keratotic patches over areas of congestion. (B and C) Histopathology revealed necrotic exudates in tissue stroma crowded with septate “spaghetti-like” fungal filaments branching at ∼45°, interspersed with shreds of vocal cord squamous epithelium (hematoxylin–eosin; 400×).

Indirect laryngoscopy and subsequent fiber-optic laryngoscopy revealed inflamed vocal cords with impaired mobility, covered with dirty-white necrotic debris resembling keratotic patches over areas of congestion. The vestibular folds were edematous (Fig. 1A). There was no palpable cervical lymph-node. Stigmata of healing or active infection, such as scar, sinus, or fistula were absent in the neck. Chest X-ray showed no evidence of active or healed tuberculosis. Routine hematologic investigations were unremarkable; she was non-diabetic and seronegative for HIV. With a provisional diagnosis of glottic malignancy, she was scheduled for microlaryngeal evaluation under general anesthesia with a plan for biopsy. It was revealed at this stage that the lesion resembling early malignancy or keratosis was actually areas of leukoplakic patch, which could be easily scraped off as a creamy layer, leaving a raw undersurface. Fungal staining of scrapings showed branching septate hyphae, morphologically resembling *Aspergillus*. Histology from vocal cord tissue samples revealed necrotic exudates in stroma crowded with septate “spaghetti-like” fungal filaments branching at ∼45°, interspersed with shreds of squamous epithelium (Fig. 1B and C). Culture reports corroborated the histology findings, confirming the growth as *Aspergillus fumigatus*.

A retrospective medical history based on the clinico-histologic findings revealed that the patient had no bronchial asthma or any sort of allergy. Further investigations were directed at any co-morbid, contributory factors that could have led to a transient immunodeficiency. However, she had no history of intake of inhalational corticosteroids or cytotoxic drugs, nor any exposure to radiation. That she was non-diabetic was known from the routine pre-operative check-up. Subsequent investigations failed to show any focus of fungal infestation in the body. This included the tracheo-bronchial tree, where flexible bronchoscopy and subsequent culture from the broncho-alveolar lavage were unremarkable, and the paranasal sinuses, which showed no evidence of infection on diagnostic nasal endoscopy and imaging. A diagnosis of primary laryngeal aspergillosis was reached, and the patient was offered oral itraconazole (300 mg/day) for three weeks. Fiber-optic laryngoscopy performed ten days following therapy showed her vocal cords to be edematous, but without any white patches or debris. She was followed up every two months. At six months, her voice had returned to normal, with no residual lesion.

## Discussion

*Aspergillus* sp. are ubiquitous, saprophytic fungi that grow on soil and decaying matter. However, they also result in opportunistic infections (sinusitis, bronchitis, allergic bronchopulmonary aspergillosis, aspergilloma, invasive aspergillosis) whose severity depends upon the virulence of the species (*A. fumigatus*, *A. flavus*, and *A. niger*) and on the host's immunity.^1^ The inhaled spores deposit on the mucosa of the paranasal sinuses, larynx, and the tracheobronchial tree, and the dark airway cavities favor their growth as hyphae. There they colonize or invade deeper tissues, producing symptoms when host immunity wanes. Laryngotracheobronchial and pulmonary aspergilloses therefore represent a group of the dreaded complications of immunocompromization. They might rarely be seen in immunocompetent individuals as well. However, primary aspergillosis restricted to the larynx in a non-immunocompromized subject is truly rare.^2^ A thorough search in the PubMed/MEDLINE, LILACS, and SciELO databases revealed only 27 cases in the English literature (Table 1).

**Table 1:** Primary laryngeal aspergillosis in immunocompetent individuals reported in English-language indexed literature.

Sl. no.	Citations	Age (years)/sex	Presentation	Associated factors	Initial diagnosis
1.	Present case; 2015	45/F	Hoarseness of voice	None	Malignancy
2.	Gangopadhyay M, Majumdar K, Bandyopadhyay A, Ghosh A. Invasive primary aspergillosis of the larynx presenting as hoarseness and a chronic nonhealing laryngeal ulcer in an immunocompetent host: A rare entity. Ear Nose Throat J. 2014;93:265–8.	42/M	Hoarseness of voice, fever, cough with expectoration	Smoking, vocal abuse	Malignancy
3.	Al-Ogaili Z, Chapeikin G, Palmer D. Primary aspergillosis of bilateral laryngoceles. Case Rep Med. 2014;2014:384271.	77^a^/F	Difficulty in swallowing and talking, hoarseness of voice	Smoking, use of inhaled corticosteroids for asthma	Lymphoma
4.	Doloi PK, Baruah DK, Goswami SC, Pathak GK. Primary aspergillosis of the larynx: a case report. Indian J Otolaryngol Head Neck Surg. 2014;66(Suppl. 1):326–8.	35/F	Hoarseness of voice, cough	None	Keratosis laryngis
5.	Ran Y, et al. 2013^5^	23/F	Hoarseness of voice, severe paroxysmal cough, tachypnea	Oral sex	None
6.	Sundarray C, Panda S, Ray R. Primary vocal cord aspergillosis in a non-immunocompromised host. J Indian Med Assoc. 2011;109:200.	NA	NA	NA	NA
7.	Ran Y, et al. 2011^2^	30/F	Hoarseness of voice, preceded by an episode of common cold (fever, headache, cough), later associated with vocal fatigue, expectoration, and occasional vomiting	Vocal abuse, oral antibiotics (ampicillin, cefixime), repeated intra-laryngeal injection of dexamethasone	Laryngitis
8.	Liu YC, et al. 2010^4^	30^a^/F	Hoarseness of voice	Vocal abuse, true vocal cord cyst	None
		32/F		Vocal abuse, therapy with broad-spectrum antibiotics	
9.	Ran Y, et al. 2008^3^	36/F	Hoarseness of voice, vocal fatigue	Systemic antibiotic (penicillin, cefotaxime) and dexamethasone therapy for rhinitis and asthma	None
10.	Wittkopf J, Connelly S, Hoffman H, Smith R, Robinson R. Infection of true vocal fold cyst with *Aspergillus*. Otolaryngol Head Neck Surg. 2006;135:660–1.	62/F	Hoarseness of voice	True vocal fold cyst (? aspergilloma)	NA
11.	Ogawa Y, et al. 2002^1^	73^a^/M	Hoarseness of voice	History of radiotherapy for laryngeal squamous cell carcinoma; history of diabetes^b^	Malignancy
12.	Dean CM, Hawkshaw M, Sataloff RT. Laryngeal aspergillosis. Ear Nose Throat J. 2001;80:300.^c^	17/F	Hoarseness of voice, vocal fatigue	None	NG
13.	Fairfax AJ, et al. 1999^7^^,^^c^	75^d^/M	Hoarseness of voice ultimately leading to aphonia	Long term use of inhalational steroid (fluticasone) through diskhaler, history of chronic smoking for 40 years, past history of carcinoma prostrate treated with bilateral orchidectomy	None
14.	Beust L, Godey B, Le Gall F, Grollier R, Le Clech G. Primary aspergillosis of the larynx and squamous cell carcinoma. Ann Otol Rhinol Laryngol. 1998; 107(10 Pt 1): 851–4.	53/M	Hoarseness of voice, respiratory distress	Radiotherapy for squamous cell carcinoma larynx	None^f^
		64/M			
15.	Nong D, et al. 1997^6^	30–40^f^/4M 4F	Hoarseness of voice leading to aphonia, mild sore throat, occasional cough (in severe cases)	None	Acute laryngitis, tuberculosis, malignancy
16.	Benson-Mitchell R, Tolley N, Croft CB, Gallimore A. Aspergillosis of the larynx. J Laryngol Otol. 1994;108:883–5.	62/M	Hoarseness of voice	None	Malignancy
17.	Kheir SM, Flint A, Moss JA. Primary aspergillosis of the larynx simulating carcinoma. Hum Pathol. 1983;14:184–6.	50/M	Hoarseness of voice	Chronic obstructive pulmonary disease	Malignancy
18.	Ferlito A. Primary aspergillosis of the larynx. J Laryngol Otol. 1974;88:1257–63.	76/M	Hoarseness of voice	None	NA
19.	Rao PB. Aspergillosis of larynx. J Laryngol Otol. 1969;83:377–9.	48/M	Hoarseness of voice	None	NA

In most cases, unless otherwise mentioned, itraconazole was the preferred anti-fungal agent.

a Surgery was the mainstay of treatment [excision of laryngoceles (serial no. 3), excision of vocal cord cyst (serial no. 8), CO_2_ laser cautery (serial no. 11)].

b According to the authors, prior radiation exposure was the more probable contributory factor for the laryngeal aspergillosis in this patient rather than diabetes.

c These citations have been retrieved as cross-references from the articles obtained following the search strategy described in the text.

d Treated with amphotericin lozenges.

e The lesions acted as harbingers of recurrence of the laryngeal cancer.

f Treated with amphotericin B (one patient), ketoconazole (three patients), and itraconazole (four patients). NA, not available; NG, not given; M, male; F, female.

Little is known about the etiopathogenesis of primary laryngeal aspergillosis in immunocompetent subjects, primarily because of limited documentation, which are mostly single-case reports. Aspergillosis is essentially opportunistic, and host immunity is the key in production of clinical disease rather than the virulence of the fungus,^1^, ^3^ although it is not clear how.^4^ Besides the known states of immunocompromization (Table 2; Group A), it may affect “apparently healthy” subjects, probably because a person “asymptomatic” at the point of contracting the disease might not be fully immunocompetent; rather, she/he could be in a transient phase of waning of immunity. This could be possible in some given conditions (Table 2; Group B). Almost all of them are systemic factors altering host immunity, except for inhaled steroids in powder form, and prior exposure to irradiation and LASER as part of the treatment protocol for laryngeal carcinoma.

**Table 2:** Suggested etiologic factors for primary laryngeal aspergillosis.

Group A	Conditions leading to an immunocompromized state	• Acquired immunodeficiency syndrome • Uncontrolled diabetes • Malignancy (especially hematologic) • Severe aplastic anemia • Prolonged neutropenia • Long-term systemic steroid intake • Active tuberculosis • Transplant recipients • On cytotoxic drugs • Chronic liver disease • Chronic debilitating illness • Terminal illness • Prolonged hospitalization • On parenteral nutrition

Group B	Conditions leading to transient systemic/local immunodeficiency in an individual who can be otherwise asymptomatic at the time of contracting the disease	• Human immunodeficiency virus infection • Diabetes under control • Oral steroid spray (powder form) and improper mouthwash • Prior irradiation • Treatment with LASER • Past history of tuberculosis • Alcoholism • Chronic obstructive pulmonary disease • On dialysis • Future recurrence of laryngeal cancer • Use of broad spectrum antibiotics

Group C	Associated conditions with only hypothetical explanation	• Vocal abuse • True vocal cyst • Oral sex • Smoking • Occupation (field work, farming, carpentry) • Laryngocele

**Figure 2:**
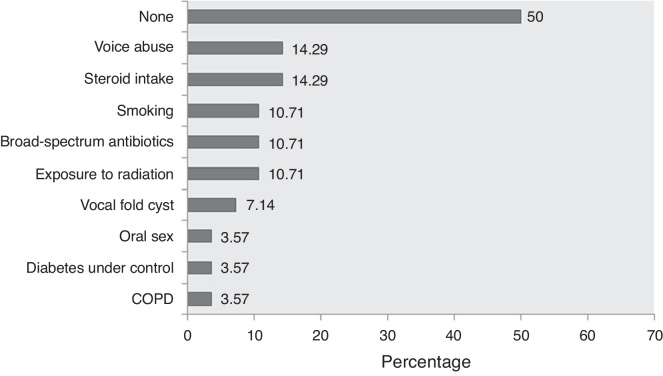
Proportionate involvement of the different factors associated with primary laryngeal aspergillosis in immunocompetent subjects (expressed in percentages). Note that in 50% of the patients, no contributory factor could be elicited. (Data were unavailable in one patient.) COPD, chronic obstructive pulmonary disease.

Besides, there are some “local” or “laryngo-tropic” factors (Table 2; Group C) whose true role in the pathogenesis remains conjectural. Many patients had history of voice abuse (∼14%), smoking (∼11%), and radiation exposure (∼11%) (Fig. 2; Table 1). It has been postulated that they cause microtrauma to the vocal fold ultrastructure, albeit in different ways, and alter the protective physiology, precipitating fungal infection.^2^ The local microbial environment is also altered by broad-spectrum antibiotics. Primary laryngeal aspergillosis has been noted in patients with bronchial asthma who chronically use inhalational steroids in powder form (Table 1). The powder granules deposit over the laryngeal epithelium and decrease local defense. Ran et al. have recently implicated the practice of oral sex among women as a potential etiology.^5^ In fact, there has been a remarkable increase in female preponderance from the overall 1.08 to 5 in the last 14 years (Table 1). The average age of presentation has also decreased – almost all women were in the reproductive age group. The reason for this skew in favor of women and younger age is not clear, but it would perhaps be too early to conclude any definitive role of oral sex in primary laryngeal aspergillosis.

Interestingly, 50% of immunocompetent subjects with primary laryngeal aspergillosis had no identifiable contributory factors that could have somehow decreased or altered the protective laryngeal physiology (Fig. 2; Table 1). Consequently, the diagnosis is not straightforward. In most patients, including the present one, the condition has been mistaken clinically as malignancy (including lymphoma) and keratosis laryngis. In the largest series published, none had a correct pre-operative diagnosis.^6^ The current patient presented with hoarseness and had irregular leukoplakic patches over the vocal cords – the typical presentation of primary aspergillosis limited to the larynx with variable subglottic and supraglottic extensions. However, it can also manifest as ulcerative plaques, as/within vocal cysts, and even within bilateral laryngoceles (Table 1), adding to the clinical dilemma.

Histopathology is essential for correct diagnosis; it demonstrates the septate hyphae with characteristic dichotomous branching at 45°.^2^, ^4^ Culture patterns (sabouraud dextrose agar at 28 °C), and presently gene extraction through a pre-formed “DNA-kit” and subsequent amplification by polymerase chain reaction, followed by sequencing in specialized laboratories provide for the species of *Aspergillus* involved.^2^, ^3^, ^4^, ^5^ Decision regarding administration of systemic antifungals is controversial, as aspergillosis is chiefly colonizing rather than invasive. However, histologic evidence of invasion has been demonstrated in immunocompetent subjects presenting with hoarseness.

The situation is akin to fungal rhinosinusitis when systemic antifungals are preferentially administered only when there is osseo-neurovascular involvement. Nevertheless, in primary laryngeal aspergillosis, oral itraconazole for three-to-four weeks has been the standard treatment in most published reports irrespective of invasion.^2^, ^3^, ^4^, ^5^ Use of ketoconazole and amphotericin B lozenges has been mentioned in few earlier reports,^6^, ^7^ but itraconazole, with fewer side-effects and satisfactory outcome, is presently favored. Data on long-term follow-up is lacking, but to date there has been no recurrence following complete treatment with systemic antifungals.

**Figure 3:**
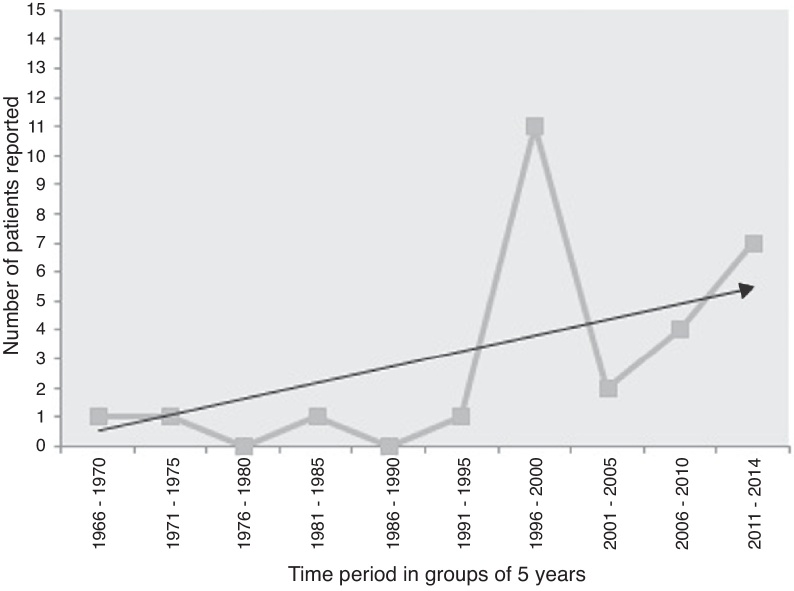
Time-trend of the number of cases of primary laryngeal aspergillosis reported in immunocompetent patients in the last five decades. The linear black line with arrow-head represents the trend-line. There has been a significant increase in reporting, especially after 1995, although eight of the 11 patients reported in the time-period of 1996–2000 were from a case-series that spanned ten years. Nevertheless, the elevation of the trend-line with time is remarkable. Primary laryngeal aspergillosis is now a disease to look for in symptomatic immunocompetent individuals. (*n.b.*: the present patient has been included in the 2011–2014 group).

Lack of definite guidelines for clinical diagnosis due of the rarity of the disease might have resulted in under-reporting, but presently primary laryngeal aspergillosis in immunocompetent subjects should be considered an “emerging disease entity.” Analysis of the frequency of cases in the five-year-wise split since its first documentation shows that there has been a steep rise of the trend-line (Fig. 3). Therefore, it might not be as uncommon as generally considered. Although the host-pathogen interaction in causation of the disease is still unknown, primary laryngeal aspergillosis currently represents an entity that present-day otolaryngologists are expected to encounter more often in non-immunocompromized individuals.

## Conclusion

The incidence of primary laryngeal aspergillosis in immunocompetent individuals has been steadily rising in the last few years. An easily removable white patch (leukoplakia), a small slough-covered ulcer, or a vocal nodule in a healthy patient presenting with hoarseness with no apparent exposure to any known immunomodulatory agent/environment should arouse suspicion of primary aspergillosis. Etiologic factors are often difficult to elicit, and immunity might not play a definitive role in the causation. However, knowledge of the suggested contributory factors with a high index of suspicion would help clinicians direct their work-up accordingly to exclude the differentials, thereby aiding to detect this potentially curable disease in time, irrespective of the status of the host's immunity.

## Conflicts of interest

The authors declare no conflicts of interest.
